# Study on the Microstructure and Corrosion Behavior of Dissimilar Aluminum Alloy Welded Joints Formed Using Laser Welding

**DOI:** 10.3390/ma17235968

**Published:** 2024-12-05

**Authors:** Suojun Zhang, Xiaozhen Liu, Shuwan Cui, Hongchen Li, Ganli Mo, Hao Li, Hongfeng Cai

**Affiliations:** 1 School of Mechanical and Automotive Engineering, Guangxi University of Science and Technology, Liuzhou 545006, China; m15176111700@163.com (S.Z.); 20230102018@stdmail.gxust.edu.cn (X.L.); lihc15946575739@163.com (H.L.); 20230103051@stdmail.gxust.edu.cn (G.M.); 20230103030@stdmail.gxust.edu.cn (H.L.); 2 Dongfeng Liuzhou Automobile Co., Ltd., Liuzhou 545005, China; liuxiaozhen955@gmail.com; 3 Guangxi Earthmoving Machinery Collaborative Innovation Center, Liuzhou 545006, China

**Keywords:** dissimilar aluminum alloys, laser welding, microstructure, electrochemical corrosion

## Abstract

This study investigates the evolution mechanisms and electrochemical corrosion behavior of laser-welded joints (WJs) between 6063 and 6082 dissimilar aluminum alloys under varying welding powers. The analysis focused on the microstructure of the weld metal zone (WMZ), its grain boundary (GB) features, and its electrochemical corrosion properties. Data from the experiments indicate that a higher laser power (LP) leads to an increase in grain size within the WMZ. At an LP of 1750 W, the weld surface exhibits the poorest corrosion resistance, while other parameters show a relatively better resistance. Additionally, electron backscatter diffraction tests indicate that the high-angle GB fraction on the 6063-T6 side of the heat-affected zone exhibits a substantially reduced measurement compared to the 6082-T6 side. The corrosion form in the WMZ is intergranular, with energy-dispersive spectroscopy (EDS) scans revealing that the poor corrosion resistance is primarily due to the presence of a large amount of Mg_2_Si phase.

## 1. Introduction

Aluminum alloys in the 6xxx series are distinguished by their low density, excellent formability, excellent mechanical properties, and resistance to corrosion, making them extensively applied in engineering materials for automotive, marine, and aerospace applications [1,2,3,4]. Meng et al. [5] highlighted that dissimilar welding can leverage the advantages of two different metals while reducing costs and saving energy. Dissimilar aluminum alloy laser welding exhibits an excellent performance and is commonly used in various applications, such as shipbuilding and lightweight automotive design. However, welding different alloys can lead to the formation of scattered intermetallic phases with diverse compositions at the weld interface, impacting corrosion resistance [6]. To further improve the performance of dissimilar aluminum alloy welds, it is essential to investigate the mechanisms behind microstructural changes and the corrosion resistance of dissimilar aluminum alloy laser welds.

Currently, significant studies have focused on the electrochemical corrosion characteristics of alloy welds. Lu et al. [7] examined how microstructural variations affect the corrosion performance of dissimilar AA7050-AA2024 aluminum alloy friction stir welds. Their findings show that weld corrosion is affected by macroelectrochemical coupling and adjacent intergranular corrosion. Shao et al. [8] examined the corrosion characteristics of 2219 aluminum alloy friction stir welds and found that a lower content of precipitate phases can reduce the effect of galvanic corrosion, as determined through a microstructural analysis. Ma et al. [9] evaluated how intermetallic compound thickness and morphology affect the corrosion performance of dissimilar aluminum alloy and steel metal joints. They observed that exceeding a heat input of 652 J/cm results in the formation of Fe-Al-Si ternary intermetallic compounds between the Fe-Al binary compound and the weld seam, which increases galvanic corrosion. Ma et al. [10] studied the galvanic corrosion of AA5052 aluminum alloy/304 stainless steel joints using zinc-based filler metals. Their macro-scale corrosion analysis revealed that the weld seam had the highest corrosion rate. Dong et al. [11] achieved welds with a superior corrosion performance compared to the base material by using a WC-W composite welding tool for friction stir welding of SUS301L stainless steel. They found that a higher austenite content enhanced corrosion resistance. Additionally, a shorter holding time at high temperatures prevented the decomposition of austenite into harmful σ phases, leading to a 0.078 V rise in the pitting corrosion potential of the joint. Gu et al. [12] investigated the microstructure and electrochemical corrosion properties of S355 steel welds subjected to ultrasonic impact treatment. The ultrasonic processing significantly refined the grain structure of the welds and enhanced their electrochemical corrosion resistance. Li et al. [13] prepared X80 steel mock welds with two different areas and discovered that the galvanic corrosion behavior of the welds was impacted by the dimensions of the heat-affected zone and the WMZ. An enhanced corrosion resistance of the weld joint is observed with a higher ratio of the WMZ to the heat-affected zone. Li et al. [14] examined the mechanisms behind corrosion fatigue failure in high-strength steel welds and identified that the primary cause of fracture was micro-galvanic corrosion between the coarse-grained heat-affected zone (CGHAZ) and the weld metal (WM). Xu et al. [15] explored how microstructure affects the corrosion behavior in dissimilar metal welds between copper and 316L stainless steel. They noted that the WMZ was clearly separated into Fe-rich and Cu-rich areas, with γ-Cu and ε-Cu phases predominating in each, respectively. The intense galvanic corrosion between these two phases ultimately led to the failure of the weld joint. Ma et al. [16] examined the corrosion characteristics and transformation mechanisms of aluminum alloy and galvanized steel welds. They discovered that as the zinc-rich phase was depleted, the joint’s corrosion mode shifted from macro-galvanic to localized corrosion, driven by incomplete corrosion product films and secondary phases like Al-Si-Mn-Fe, Al_3_Fe_2_Si_3_, Al_3_Fe_2_Si, Fe_2_Al_5_, and Fe_4_Al_13_. Pan et al. [17] investigated the corrosion performance of aluminum/galvanized steel joints exposed to a salt spray environment, comparing resistance spot welding (RSW) and self-piercing riveting (SPR) methods. In the RSW joints, severe pitting was observed in the aluminum coupling areas, while the corrosion in the steel coupling areas was less severe due to the protective zinc coating. In the SPR joints, the presence of smaller gaps resulted in an increased accumulation of OH^−^ ions, which passivated the aluminum in the coupling zone and resulted in a lower corrosion rate for the aluminum. Ahmad et al. [18] studied the fatigue and electrochemical corrosion properties of dissimilar welds between Alloy 617 and 12Cr steel, employing both overlay and non-overlay welding methods. By comparing the performance of the welds produced by these methods, they concluded that overlay welding enhanced both the corrosion resistance and fatigue durability of the joints between Alloy 617 and 12Cr steel.

Scholarly research shows that understanding the mechanisms of microstructural evolution and electrochemical corrosion in metal welds is essential for extending their service life. At present, there is a scarcity of literature regarding the effects of welding process parameters on various aspects of aluminum alloy laser welding. Therefore, a comprehensive study of the microstructural evolution mechanisms and electrochemical corrosion behavior of 6063/6082 dissimilar aluminum alloy laser welds is imperative. This study will explore the microstructure and corrosion mechanisms of laser welds between the dissimilar aluminum alloys 6063 and 6082, contributing to the advancement of aluminum welding technology.

## 2. Materials and Methodology

The aluminum alloys used in this study were 6063-T6 and 6082-T6, each with a thickness of 2.5 mm. Table 1 [19] depicts the chemical compositions of these alloys. The laser welding laser type is Nd:YAG laser. Laser welding was selected to join the aluminum alloys, using the parameters outlined in Table 2 [19].

**Figure 1:**
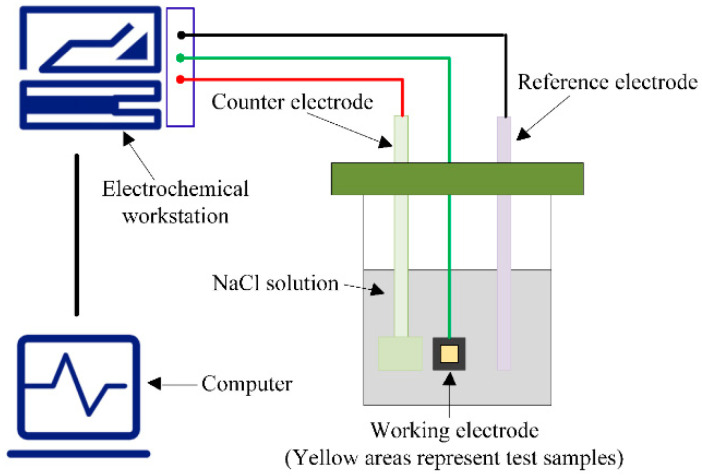
Schematic diagram of electrochemical testing.

After welding, the joints were subjected to electrical discharge machining to obtain the required microstructural samples and electrochemical corrosion test specimens. Subsequently, the cut samples were subjected to grinding and polishing. The working electrodes for the base metal (BM) and the laser weld zone of the dissimilar aluminum alloys 6063 and 6082 were prepared using a cold embedding technique. The experiments were conducted at room temperature (25 °C) using a three-electrode electrochemical corrosion system, with the weld joint as the working electrode, a platinum electrode as the counter electrode, and a saturated calomel electrode as the reference electrode. A 3.5% NaCl solution was used as the working medium. Prior to testing, the samples were immersed in the working medium for 60 min, and measurements were taken after the open-circuit corrosion potential stabilized, with a scanning rate of 1 mV/s. Initially, the open-circuit potential was measured using an electrochemical workstation, and the measurement range for the polarization curves was chosen according to the open-circuit potential readings. The polarization curves were then chosen accordingly. Figure 1 presents a schematic representation of the electrochemical testing process. After measurements, the samples were carefully stripped (with the test surfaces protected), and an examination of the test surfaces was conducted using techniques like scanning electron microscopy and X-Ray photoelectron spectroscopy.

## 3. Microscopic Structure Analysis

Table 3 presents the joint morphology obtained from welding experiments at welding speeds of 10 mm/s and 15 mm/s, with laser powers of 1500 W, 1750 W, and 2000 W. At a welding speed of 10 mm/s the welds exhibited good formation on both the front and back sides for all of the laser power levels. At a laser power of 1750 W, the weld formation was optimal compared to the other power levels at the same speed. However, when the welding speed increased to 15 mm/s, only the front side displayed proper weld formation, while the back side was not fully penetrated. Nevertheless, welding defects on both the front and back sides were nearly eliminated at the other laser power levels.

**Figure 2:**
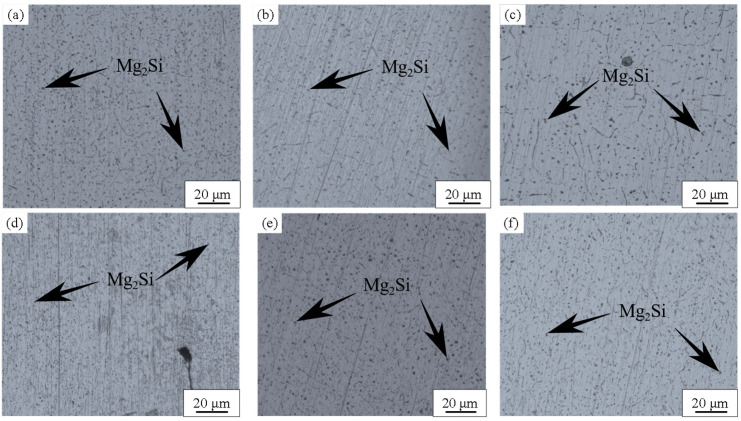
Microstructure of the central region of WMZ: (**a**) P = 1500 W and V = 10 mm/s; (**b**) P = 1750 W and V = 10 mm/s; (**c**) P = 2000 W and V = 10 mm/s; (**d**) P = 1500 W and V = 15 mm/s; (**e**) P = 1750 W and V = 15 mm/s; (**f**) P = 2000 W and V = 15 mm/s.

**Figure 3:**
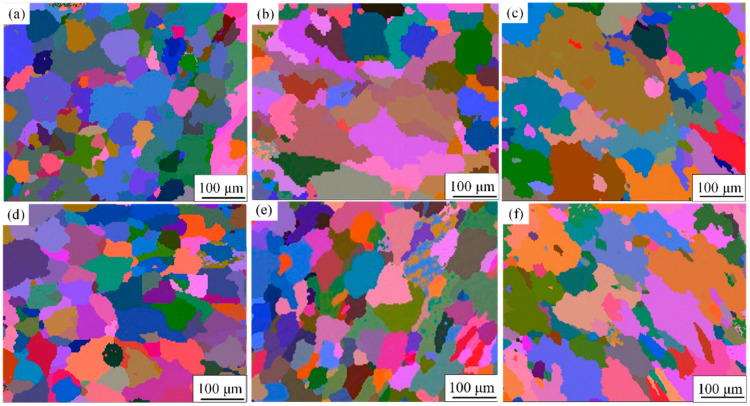
Euler diagram of the center region of WMZ: (**a**) P = 1500 W and V = 10 mm/s; (**b**) P = 1750 W and V = 10 mm/s; (**c**) P = 2000 W and V = 10 mm/s; (**d**) P = 1500 W and V = 15 mm/s; (**e**) P = 1750 W and V = 15 mm/s; (**f**) P = 2000 W and V = 15 mm/s.

Figure 2 displays metallographic images of the central region of the WMZ under different welding parameters. In this region, the microstructure consists of cellular dendrites. The small black particles are Mg_2_Si, typically present in 6xxx-series aluminum alloys [20]. At a welding speed of 10 mm/s, the appearance of cellular dendrites becomes more pronounced with increasing welding pressure. However, at a welding speed of 15 mm/s the variation in cellular dendrites under different welding pressures is less noticeable. As shown in Figure 3a,d, when the LP is 1500 W, insufficient heat input results in gaps and incomplete fusion, leading to an incomplete microstructure. At both welding speeds, as the LP increases the metallographic structure of the WMZ becomes more distinct.

Laser power and welding speed are the main factors influencing the microstructure of weld joints. As the laser power increases, the heat input during welding rises, resulting in higher temperatures in the molten pool, which increases both the penetration depth and width. An excessive heat input can slow the cooling rate, allowing grains sufficient time to grow, leading to coarser grains. As shown in Figure 3, under two different speeds, the grain size in the WZM gradually increases with the continuous increase in welding power. The higher welding speed leads to a relatively fast cooling rate of the weld metal zone, resulting in a smaller grain size. According to previous studies [21], the smaller grain size improves the tensile strength of the material. Figure 3 shows the Euler diagrams of the central WMZ region for different welding parameters. It can be observed from the figure that, at the same welding speed, the average grain size in the central WMZ region grows with increasing LP. Table 4 presents the average grain sizes in the central region of the WMZ for various welding parameters.

Previous research indicates that the grain size in the weld region correlates with heat input; a reduced heat input leads to finer grain sizes. [22]. For a welding speed of V = 10 mm/s, the average grain sizes under welding powers of P = 1500, 1750, and 2000 W are 39.45 μm, 41.42 μm, and 44.07 μm, respectively. At a welding speed of V = 15 mm/s and welding powers of P = 1500, 1750, and 2000 W, the average grain sizes are 39.42 μm, 45.74 μm, and 49.61 μm, respectively. With constant welding speed, a higher welding power results in a larger average grain size in the weld center region. The difference in average grain size at different LPs is 4.77 μm at V = 10 mm/s, and 2.60 μm at V = 15 mm/s.

**Figure 4:**
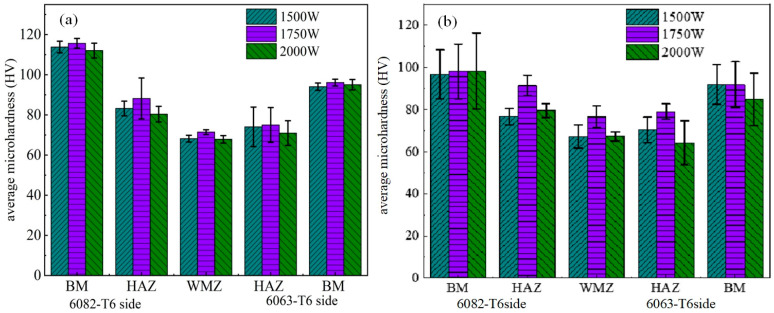
Microhardness profiles of the weld joints: (**a**) V = 10 mm/s; (**b**) V = 15 mm/s.

**Figure 5:**
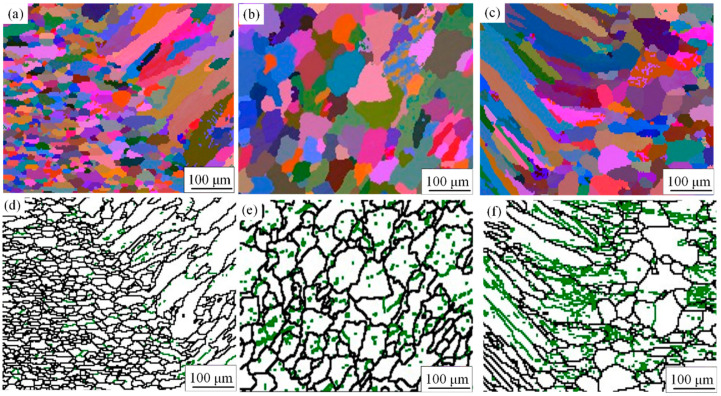
Euler diagrams at V = 15 mm/s and P = 1750 W: (**a**) near the 6082-T6 side fusion line; (**b**) in the WMZ center region; and (**c**) near the 6063-T6 side fusion line. Grain boundary angle orientation difference diagrams: (**d**) near the 6082-T6 side fusion line; (**e**) in the WMZ center region; and (**f**) near the 6063-T6 side fusion line.

Previous studies have found that under high-heat-input welding conditions, the performance of the heat-affected zone (HAZ) significantly deteriorates [23]. In the HAZ, the microstructure and properties of the material change due to the heat from welding. In the HAZ, the material experiences different temperature variations, which can affect the grain size. Grain coarsening can reduce the material’s tensile strength and yield strength. Additionally, microstructural changes in the HAZ may lead to decreased corrosion resistance, impacting the durability of the weld joint and increasing the risk of failure. Figure 4a shows the microhardness profile of the weld joint at a welding speed of V = 10 mm/s, while Figure 4b presents the microhardness profile at V = 15 mm/s. It can be observed that the microhardness in the WMZ is consistently higher at a welding speed of V = 15 mm/s, compared to V = 10 mm/s. Yang et al. [24] determined that dendrite growth rate is affected by the solidification rate and temperature gradient. Figure 5 displays the Euler diagrams and corresponding GB distribution maps for the 6063-T6 alloy near the fusion line, the WMZ, and the 6082-T6 alloy near the fusion line under a welding speed of 15 mm/s and an LP of 1750 W. As shown in Figure 5a–c, the microstructure at the weld center is characterized by cellular dendrites, while coarser columnar grains are present near the fusion line on both sides. The higher normal temperature gradient on either side of the fusion line is greater than that in the weld center. This difference results in slower solidification rates. The temperature gradient-to-solidification rate ratio becomes relatively large. Consequently, this reduces the degree of composition undercooling. Additionally, the weld metal solidifies starting at the fusion line. This is where the joint begins to crystallize. As a result, the microstructure changes from coarse columnar grains to cellular dendrites. This transition occurs from the edge of the fusion line towards the weld center.

Cui et al. [25] indicated that in polycrystalline materials, the Grain Boundary Misorientation Angle Distribution (GBMAD) influences the mechanical properties of the material. According to the misorientation angle of GBs, GBMAD can be categorized into low-angle GBs (LAGB) (2° ≤ θ ≤ 10°) and high-angle GBs (HAGB) (θ ≥ 15°). Figure 5d–f show the GBMAD corresponding to Figure 5a–c. Black lines represent HAGB, while green lines indicate LAGB. In specific circumstances, a higher proportion of HAGB contributes to a gradual improvement in impact toughness of the material. At the same time, Song et al. [26] noted that low-angle grain boundaries exhibit better intergranular corrosion and stress corrosion resistance, while high-angle grain boundaries are more susceptible to intergranular corrosion. The results indicate that the proportion of HAGB is a key criterion for assessing the impact toughness of the WMZ. Comparisons show that the greatest amount of LAGB is found in the heat-affected zone (HAZ) close to the fusion line on the 6063-T6 side. It can be inferred that the area of mechanical property decline in the welded joint is located in the heat affected zone near the fusion line on the side of 6063-T6, and the area of corrosion property decline is located in the heat affected zone near the fusion line on the side of 6082-T6.

## 4. Electrochemical Corrosion Behavior Analysis

**Figure 6:**
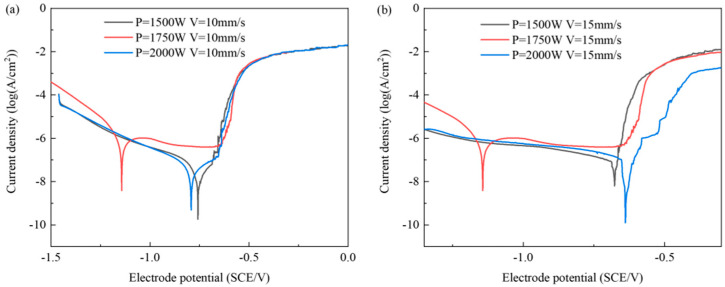
Polarization curves are plotted. (**a**) V = 10 mm/s; (**b**) V = 15 mm/s.

As shown in Figure 2, when the LP is 1750 W, the proportion of Mg_2_Si strengthening phases in the weld zone is relatively high, leading to a decrease in potential [16]. Figure 6a illustrates the electrochemical corrosion polarization curves of the weld joint surfaces under different LPs at a welding rate of 10 mm/s. Notably, when the LP is set to P = 1750 W, the polarization curve displays a distinct pattern compared to the other two LP settings, characterized by a larger passivation region. As shown in Table 5, the corrosion potential values for LPs of 1500 W, 1750 W, and 2000 W are −0.758 V, −1.143 V, and −0.863 V, respectively. Among these, the highest corrosion potential is observed at P = 1500 W, while the lowest corrosion potential is observed at P = 1750 W. Based on corrosion potential assessments, it is evident that the WMZ surface exhibits the best corrosion resistance at a welding rate of 10 mm/s and LP of P = 1500 W, whereas the worst corrosion resistance is observed at P = 1750 W under the same welding speed. At a welding rate of 10 mm/s, as the welding power increases, the self-corrosion current densities are 2.2825 × 10^−8^ A/cm^2^, 7.5831 × 10^−7^ A/cm^2^, and 1.0473 × 10^−8^ A/cm^2^, respectively. A higher self-corrosion current density indicates a faster corrosion rate and poorer corrosion resistance. Therefore, at a welding rate of 10 mm/s and an LP of P = 1750 W, the WMZ surface exhibits the poorest corrosion resistance [17,27].

Figure 6b depicts the polarization curves for various LPs at a welding rate of 15 mm/s. As in Figure 6a, the polarization curve for the WMZ at P = 1750 W exhibits a distinctly different profile compared to the other two LP settings, showing the lowest corrosion potential and the highest corrosion rate. With increasing LP, the corrosion potentials are −0.639 V, −1.143 V, and −0.677 V, respectively, while the self-corrosion current densities are 1.4479 × 10^−8^ A/cm^2^, 8.5428 × 10^−7^ A/cm^2^, and 1.6405 × 10^−8^ A/cm^2^. Analysis of the corrosion potential and self-corrosion current density indicates that, at a welding rate of 15 mm/s and an LP of P = 1750 W, the WMZ exhibits the highest corrosion rate and the poorest corrosion resistance. Comparative analysis reveals that, regardless of the welding speed, the WMZ exhibits the poorest corrosion resistance when the LP is set to P = 1750 W.

At both welding speeds, when the LP is set to P = 1750 W, the surface concentration of Mg exceeds that of Si. This phenomenon is related to the initial electrochemical potentials of the Al matrix, the Mg_2_Si strengthening phase, and the Si elements. Specifically, Si elements have the most positive initial electrochemical potential, the Mg_2_Si strengthening phase has the most negative initial potential, while the Al matrix’s initial potential is intermediate between the two. A microcell forms between the high- and low-potential regions, leading to the preferential dissolution of the high potential material, which acts as the anode. Because of the high reactivity of Mg within the Mg_2_Si phase, it preferentially dissolves, resulting in the release of more Mg and Si elements. The accumulation of the less reactive Si element causes the potential of the Mg_2_Si phase to shift positively. When the concentration of Si reaches a certain level, a shift from cathodic to anodic behavior occurs. Ultimately, a microcell forms between the Mg_2_Si reinforcement phase and the surrounding Al matrix, with the Al matrix dissolving as the anode.

**Figure 7:**
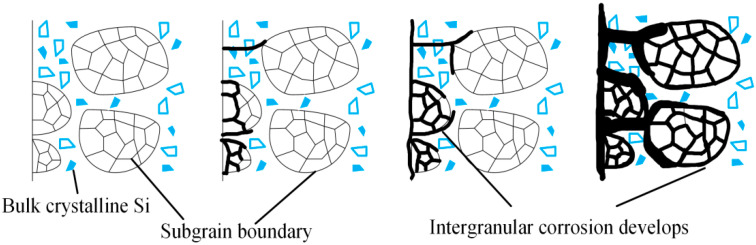
Schematic diagram of intergranular corrosion.

**Figure 8:**
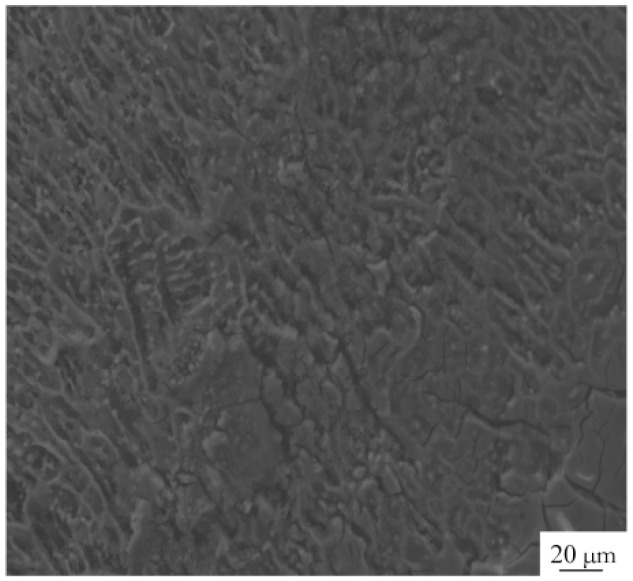
Test sample surface.

In Figure 3, the black spots represent the Mg_2_Si precipitate phase. Previous studies have found that during the welding process of aluminum alloys, the WMZ precipitates Mg_2_Si metal strengthening phases, which form micro-galvanic couples with the base metal (BM) phases, thereby reducing the corrosion performance of the weld [16]. A microstructural analysis reveals that the primary microstructural constituents in the BM, and the WMZ of the WJ, feature an Al matrix with the Mg_2_Si strengthening phases precipitated along the GBs. Figure 7 illustrates the schematic process of intergranular corrosion, where precipitates at the GBs form an enriched layer, leading to a potential difference across the enriched layer and the matrix. During the electrochemical corrosion process, when the corrosive solution contacts the surface, the region with the lower potential acts as the anode, creating a micro-cell with the cathode. This results in the formation of continuous active anode corrosion pathways along the GBs, causing the precipitates distributed at the GBs to preferentially dissolve in the corrosive medium. Corrosion propagates along the GBs and, over time, the intergranular corrosion cracks gradually coalesce, extending from the GBs into the grain interior and penetrating deeper into the specimen, ultimately forming intergranular corrosion cracks of significant depth. Figure 8 illustrates the sample surface examined with a Scanning Electron Microscope (SEM) following electrochemical corrosion, revealing that corrosion is concentrated along the grain boundaries, leading to intergranular corrosion.

**Figure 9:**
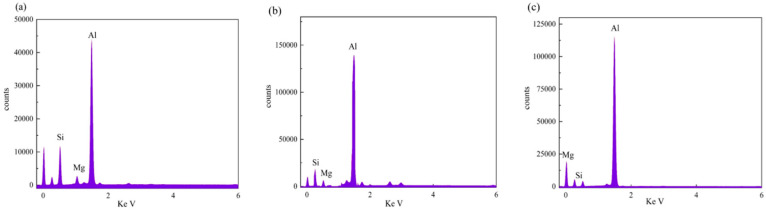
Energy spectra corresponding to Table 3: (**a**) 1500 W; (**b**) 1750 W; (**c**) 2000 W.

To elucidate the elemental distribution patterns on the WMZ surface after electrochemical corrosion, an EDS analysis was performed on the corroded area. Surface scanning techniques were employed to analyze the WMZ of the WJs. Table 6 provides the elemental mapping of Al, Mg, and Si under different LP settings at a rate of 10 mm/s for welding. Across all LP conditions, the concentration of Al is significantly higher compared to the other two elements. Figure 9 presents the EDS spectra corresponding to different welding power settings. Comparative analysis reveals that at an LP of P = 1750 W, the surface of the WMZ shows the densest and most abundant distribution of Mg elements after electrochemical corrosion, while the distribution of Si elements is sparse and minimal. At this LP, the concentrations of Mg and Si elements differ significantly from those at the other two LP settings.

**Figure 10:**
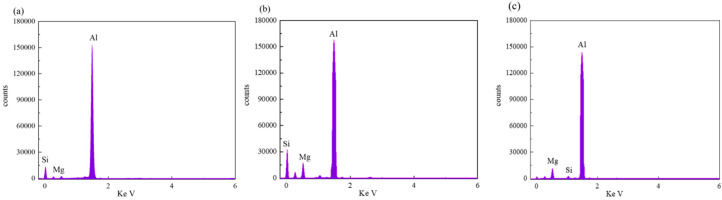
Energy spectra corresponding to Table 4: (**a**) 1500 W; (**b**) 1750 W; (**c**) 2000 W.

Table 7 presents the elemental mapping analysis of the weld metal area on the WJ surface after electrochemical corrosion at different LPs with a welding speed of V = 15 mm/s. The results indicate that, consistent with previous discussions, the content of Al is significantly higher than that of the other two elements in each LP group. Figure 10 presents the corresponding EDS spectra, showing the contents of Al, Mg, and Si elements. When the LP is 1500 W and 1750 W, the content of Si is higher than that of Mg. However, at an LP of 2000 W, the content of Mg exceeds that of Si.

## 5. Conclusions

In this study, experimental methods were used to investigate the effects of laser welding power and welding speed on the microstructure of the heat-affected zone (HAZ) and the electrochemical corrosion behavior of 6063/6082 aluminum alloy weld joints. The study utilized EBSD testing to analyze the microstructure of the weld joints and employed an electrochemical workstation to assess the electrochemical corrosion behavior, with Nd pulsed laser welding used for the welding process. The following findings were obtained regarding the WJ:

1. When the laser power is set to 1750 W, and the welding speed is V = 15 mm/s, the content of the precipitated Mg_2_Si phase in the weld zone is at its highest.
2. With a constant welding speed, an increase in laser power results in a larger grain size in the weld zone. At a welding speed of V = 15 mm/s, and laser power of P = 1750 W, it was observed that, on the 6063-T6 side, the number of low-angle grain boundaries is highest in the HAZ near the fusion line, whereas on the 6082-T6 side, the number of low-angle grain boundaries is lowest in the HAZ near the fusion line.
3. At both welding speeds, when the laser power is 1750 W, the corrosion potential of the weld joint surface is at its lowest.
4. Energy-dispersive spectra and elemental maps indicate that, as the welding power increases, the content of Si in the weld joint gradually decreases, while the content of Mg gradually increases.

## Figures and Tables

**Table 1:** Chemical composition of the aluminum alloys in wt%.

Alloy	Si	Mn	Mg	Cu	Zn	Ti	Fe	Al
6063	0.381	0.009	0.707	0.040	0.003	0.029	0.244	Bal.
6082	1.000	0.560	1.000	0.030	0.060	0.030	0.330	Bal.

**Table 2:** Welding parameters.

Test Number	Welding Speed (mm/s)	LP(W)	Argon Flow Rate (L/mim)
1	10	1500	20
2	10	1750	20
3	10	2000	20
4	15	1500	20
5	15	1750	20
6	15	2000	20

**Table 3:** Front and back view of welded joints.

Welding Speed (mm/s)	LP (W)	Weld Face	Weld Root
10	1500	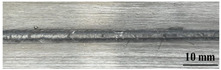	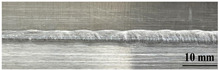
10	1750	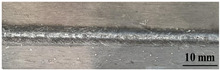	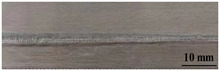
10	2000	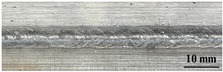	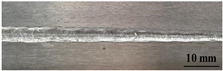
15	1500	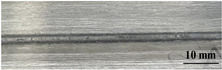	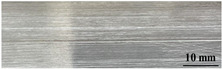
15	1750	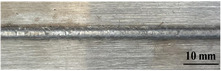	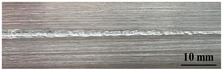
15	2000	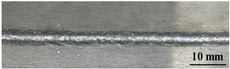	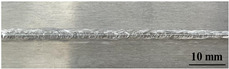

**Table 4:** Average size of grains.

Welding Speed (mm/s)	LP (W)	Average Grain Size (µm)
10	1500	39.54
10	1750	41.42
10	2000	44.07
15	1500	39.42
15	1750	45.74
15	2000	49.61

**Table 5:** Characteristic parameters of electrochemical corrosion.

Welding Speed (mm/s)	LP (W)	Corrosion Potential (V)	Self-Corrosion Current Density (A/cm^2^)
10	1500	−0.758	1.0473 × 10^−8^
10	1750	−1.137	7.5831 × 10^−7^
10	2000	−0.863	2.2825 × 10^−8^
15	1500	−0.639	1.4479 × 10^−8^
15	1750	−1.143	8.5428 × 10^−7^
15	2000	−0.677	1.64059 × 10^−8^

**Table 6:** V = 10 mm/s Layered table of corrosion surface elements.

LP	Layered Images of Al Elements	Layered Images of Mg Elements	Layered Images of Si Elements
1500 W	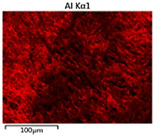	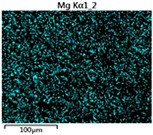	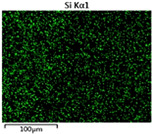
1750 W	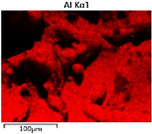	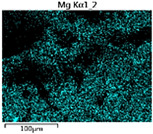	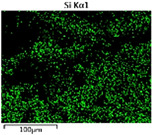
2000 W	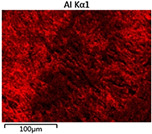	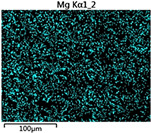	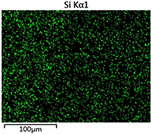

**Table 7:** V = 15 mm/s Layered table of corrosion surface elements.

LP	Layered Images of Al Elements	Layered Images of Mg Elements	Layered Images of Si Elements
1500 W	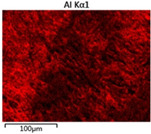	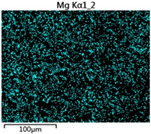	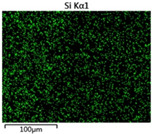
1750 W	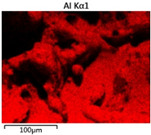	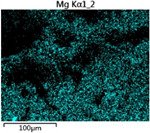	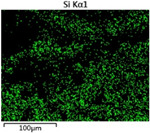
2000 W	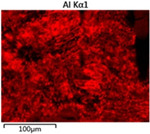	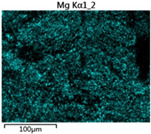	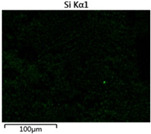

## Data Availability

The datasets generated and analyzed during the current study are not publicly available, due to the fact that the data are presented within the article, but they are available from the corresponding author on reasonable request.
